# Deformation-Aware Monte Carlo Assessment of a Bi_2_O_3_/Thermoplastic Polyurethane Shielding Surrogate Under Tensile Strain and Local Thickness Loss

**DOI:** 10.3390/polym18192363

**Published:** 2026-09-28

**Authors:** Ahmed Alharbi, Zeyad Alawaji

**Affiliations:** 1 Department of Physics, College of Science, Qassim University, Buraydah 51452, Saudi Arabia; 2 Department of Radiologic Technology, College of Applied Medical Sciences, Qassim University, Buraydah 51452, Saudi Arabia; z.alawaji@qu.edu.sa

**Keywords:** thermoplastic polyurethane, bismuth oxide, X-ray shielding, deformation, Monte Carlo, Geant4, PHITS, SpekPy, local thinning, wearable radiation protection

## Abstract

Flexible shielding polymers may undergo geometric thinning during tensile use or localized damage, but flat-sheet attenuation measurements cannot resolve the resulting spatial loss of protection. A radiation-transport surrogate comprising 60 wt% thermoplastic polyurethane and 40 wt% ${\mathrm{Bi}}_{2}$$O_{3}$ (modeled density, 1.835 g ${\mathrm{cm}}^{-3}$) was investigated using prescribed deformation, Geant4, PHITS, Phy-X/XCOM, and 60–120 kVp SpekPy spectra. This is a computational transport model, not a mechanically or experimentally validated formulation. Geant4 mass attenuation coefficients were verified against Phy-X at six energies (30–100 keV) and against XCOM at 90, 91, 92, and 95 keV across the Bi K-edge. Constant-volume uniform strain of 50% raised whole-field transmission by 14.8–32.8%; an illustrative transverse-response sensitivity quantified the dependence of thickness and attenuation on the prescribed kinematics. At 50% local thinning, whole-field transmission increased by only 5.66–15.0%, whereas 5-million-history event-based ROI scoring gave dose enhancement factors of 2.435±0.106, 1.823±0.064, 1.508±0.048, and 1.395±0.042 at 60, 80, 100, and 120 kVp, respectively. Additional 60 kVp calculations varied the defect dimensions, ROI size, and detector position, revealing the influence of spatial averaging. Beer–Lambert attenuation accounts for the primary response to prescribed uniform thinning; the Monte Carlo framework additionally resolves polychromatic scatter, spectral changes, and localized dose enhancement. These numerical results characterize idealized geometric perturbations and should not be interpreted as measurements of real garment durability or clinical dose.

## 1. Introduction

Lead remains an efficient photon shield because of its high atomic number and density, but its toxicity, mass, and limited flexibility motivate continued development of lead-free polymer composites for medical, industrial, and wearable radiation protection [1,2,3,4]. High-Z fillers such as bismuth-, tungsten-, and gadolinium-containing phases can provide substantial X-ray attenuation, while a polymer matrix contributes processability, reduced weight, and mechanical compliance [1,2,3,4].

Bismuth-containing flexible systems are particularly relevant to diagnostic X-ray protection. ${\mathrm{Bi}}_{2}$$O_{3}$ has been incorporated into flexible polymer films, elastomers, and polyurethane-based coatings [5,6,7], while TPU has been used in flexible shields containing Bi-rich metallic or multiphase fillers [8,9]. Recent studies have focused on composition, filler architecture, loading, particle size, multilayer design, and lead equivalence [5,6,7,8,9,10,11]. These developments establish the feasibility of flexible high-Z composites, but a flat-coupon measurement does not determine the consequences of prescribed stretching or local thinning. The present calculation does not model actual cracking, wear morphology, or filler redistribution.

This question is practically relevant to radiation-protective garments. Radiographic and fluoroscopic quality-assurance programs routinely inspect aprons and thyroid collars for tears and cracks because small damaged regions can compromise local protection [12,13,14]. Longitudinal inspection data show that defects are common in personal radiation protective equipment, and recent dosimetric studies have moved beyond defect area alone toward dose- and location-aware discard criteria [14,15,16]. This literature motivates a spatially resolved treatment of flexible shielding: a small defect may be strongly diluted in a whole-area transmission measurement even when the dose immediately behind the defect is substantially increased.

Attenuation also depends strongly on mass thickness along the photon path. For a nearly incompressible flexible sheet, tensile extension produces a predictable reduction in thickness; nonuniform strain, wear, or manufacturing defects can additionally produce spatially localized thickness losses. Recent reviews have emphasized the need to connect the mechanical state of polymer shielding with radiation performance [1,2]. The studies cited above motivate separating analytically predictable uniform mass-thickness changes from spatially heterogeneous shielding losses. The present work examines that distinction in a controlled transport surrogate, rather than inferring the mechanical response of a fabricated composite.

The purpose of the present work is therefore not to claim that deformation can affect shielding in principle. Instead, it establishes a reproducible deformation-aware radiation-transport framework and quantifies how much information can be lost when a flexible shield is characterized only in its undeformed state. A 60 wt% TPU/40 wt% ${\mathrm{Bi}}_{2}$$O_{3}$ computational surrogate was modeled. The workflow combines (i) attenuation validation against Phy-X, (ii) independent PHITS-Geant4 verification, (iii) an analytical Beer–Lambert reference for uniform strain, (iv) realistic 60–120 kVp spectra, and (v) high-statistics local-defect scoring. The working hypothesis is that idealized uniform strain will be dominated by the predictable reduction in mass thickness, whereas a localized defect will produce a much larger regional loss of protection than is evident from whole-field metrics.

## 2. Materials and Methods

### 2.1. Composite Model

The modeled shield was a computational surrogate consisting of 60 wt% representative TPU and 40 wt% ${\mathrm{Bi}}_{2}$$O_{3}$. For physical readability, the elemental mass fractions are reported here as C = 0.3600, H = 0.0420, N = 0.0420, O = 0.1972, and Bi = 0.3588, and the modeled composite density is reported as 1.835 g ${\mathrm{cm}}^{-3}$. The Geant4 composite density was 1.83505155 g ${\mathrm{cm}}^{-3}$ (rounded to 1.835 g ${\mathrm{cm}}^{-3}$ in the text). The empirical descriptors $C_{40}$$H_{56}$$N_{4}$$O_{13}$ for the TPU phase and $C_{280}$$H_{392}$$N_{28}$$O_{115}$${\mathrm{Bi}}_{16}$ for the combined composition used in Phy-X are elemental transport surrogates rather than molecular repeat-unit assignments to a specific commercial TPU grade. ${\mathrm{Bi}}_{2}$$O_{3}$ was assigned a density of 8.90 g ${\mathrm{cm}}^{-3}$.

The assumed neat-TPU density of 1.20 g ${\mathrm{cm}}^{-3}$ is representative of commercial grades. For example, Covestro reports 1.196 g ${\mathrm{cm}}^{-3}$ for Desmopan 487 and 1.210 g ${\mathrm{cm}}^{-3}$ for Desmopan 487 DPS 062, with reported strains at break of 556% and 460%, respectively [17,18]. Thus, the maximum 50% tensile strain examined here is well below the ultimate deformation of these example neat-TPU grades. Experimental digital-image-correlation measurements have also shown a nonlinear TPU Poisson’s ratio of approximately 0.45 during first loading, increasing toward approximately 0.48 after unloading and subsequent cycling [19]. These observations motivate a near-incompressible illustrative reference for neat TPU but do not establish the Poisson response of the filled composite.

The present 40 wt% ${\mathrm{Bi}}_{2}$$O_{3}$ composite is not assumed to retain the constitutive response of neat TPU. Rigid filler loading can modify stiffness, transverse contraction, hysteresis, permanent set, damage initiation, and interfacial void formation. No filler agglomeration, porosity, interfacial debonding, cracking, strain-induced filler migration, or constitutive damage law was introduced. Consequently, the model isolates the radiation consequence of prescribed geometric mass-thickness changes rather than predicting the mechanical response of a fabricated formulation. This distinction is central to the scope of the study and is revisited in the Limitations subsection.

The irradiation geometry and the three modeled shield configurations are shown in Figure 1.

### 2.2. Prescribed Deformation Models

The nominal shield dimensions were $L_{x,0}=L_{y,0}=10$ cm and $t_{0}=2.0$ mm. Uniform uniaxial deformation was prescribed rather than solved mechanically. A nearly incompressible sheet was assumed, with equal contraction in the transverse in-plane and through-thickness directions. For engineering strain ε and stretch ratio λ = 1 + ε, the dimensions were

(1) $$L_{x}=L_{x,0}\lambda ,\;L_{y}=\frac{L_{y,0}}{\sqrt{\lambda }},\;t=\frac{t_{0}}{\sqrt{\lambda }}.$$

Equation (1) conserves the modeled shield volume, so the density and filler concentration remain unchanged. Strains of 0, 10, 20, 30, 40, and 50% were evaluated. The resulting dimensions are summarized in Table 1.

An illustrative transverse-response sensitivity was calculated to test the kinematic assumption without claiming composite-specific mechanical validation. With an assumed effective transverse exponent ν, equal lateral stretches were prescribed as $\lambda_{y}=\lambda_{z}=\lambda^{-\nu }$. For a fixed shield mass, the corresponding volume ratio, density, and areal mass are

(2) $$\frac{V}{V_{0}}=\lambda^{1-2\nu },\;\rho \left(\lambda ,\nu \right)=\rho_{0}\lambda^{2\nu -1},\;\rho t=\rho_{0}t_{0}\lambda^{\nu -1}.$$

The parameter ν is an illustrative finite-stretch transverse exponent, not a measured or assumed constant small-strain Poisson ratio of the 40 wt% composite. Values of 0.40–0.50 bracketed plausible alternative prescribed contractions for a sensitivity test; only ν=0.50 corresponds to the constant-volume production model. In the non-volume-conserving examples, density is adjusted by mass conservation rather than held constant. At 50% strain, the predicted 60 keV uncollided Beer–Lambert response changes, as shown in Table 2. This is an analytical sensitivity calculation, not additional Geant4 material validation.

A second geometry represented spatially localized wear or nonuniform thinning. The outer sheet remained 10 × 10 ${\mathrm{cm}}^{2}$ and 2.0 mm thick, while a central 2 × 2 ${\mathrm{cm}}^{2}$ patch was reduced according to $t_{\mathrm{local}}=t_{0}\left(1-\delta \right)$, where δ was 10–50%. Thus, the patch thicknesses were 1.8, 1.6, 1.4, 1.2, and 1.0 mm for 10, 20, 30, 40, and 50% local thickness loss, respectively. The patch occupied 4% of the shield area (400 ${\mathrm{mm}}^{2}$), deliberately creating a geometry in which strong local leakage can be diluted in a whole-area average. The defect was implemented as a sharp-edged thickness step to provide a clean, reproducible transport perturbation; real wear is commonly irregular or graded, so the geometry should not be interpreted as a literal morphology or universal rejection threshold. To examine the geometric sensitivity of the local response at 60 kVp and 50% thickness loss, additional central square defects with side lengths of 1 and 4 cm were modeled, each evaluated against a fixed central 2 × 2 ${\mathrm{cm}}^{2}$ ROI. Independently, 1 and 4 cm square ROIs were evaluated with the reference 2 cm square defect. The water-front position was also varied from z=1.0 cm to z=0.5 and 5.0 cm; since the shield’s back face is at z=0.2 cm, these correspond to downstream gaps of 0.3, 0.8, and 4.8 cm. The reference 2 cm defect/ROI and z=1 cm result uses the independent 5-million-history run, whereas the other sensitivity cases used 1-million-history runs. At 50% uniform strain, the transverse dimension remains 8.165 cm, which fully covers the 6 × 6 ${\mathrm{cm}}^{2}$ incident field.

### 2.3. Geant4 Radiation-Transport Model and Scoring

Geant4 version 10.06.p02 (Geant4 Collaboration, CERN, Geneva, Switzerland) was used for the production calculations [20,21]. This release was retained because it was the established build of the validated application used for the matched PHITS and attenuation-verification workflow. Newer Geant4 11.x releases are available [22]; rather than assuming software-version invariance, the present study constrains implementation and cross-section effects by independent Phy-X/XCOM and PHITS verification. Low-energy electromagnetic transport used the Livermore physics configuration, fluorescence and Auger production were enabled, and a 0.01 mm production cut was applied.

The surrounding world material was Geant4G4_Galactic (vacuum), chosen to isolate shield transport from air attenuation and scatter. A General Particle Source emitted photons uniformly from a 6 × 6 ${\mathrm{cm}}^{2}$ rectangular plane at z=−5 cm. All source photons had the same direction vector (0,0,1), so the field was parallel and normally incident rather than divergent. The shield’s front face was positioned at z=0. A water scoring slab measuring 10 × 10 × 0.1 ${\mathrm{cm}}^{3}$ occupied z=1.0–1.1 cm. Even at the maximum 50% tensile strain, the shield width transverse to extension was 8.165 cm and, therefore, remained larger than the 6 cm beam width.

Each photon entering the water volume was recorded as a transmitted crossing. A primary photon was additionally classified as uncollided when no prior interaction had been registered and its propagation remained along +z. Total energy deposition in the 1 mm water slab was accumulated as absorbed dose per source photon. At the diagnostic photon energies considered here, this thin-water quantity is used as a local kerma-like energy-deposition surrogate for relative comparisons; it is not interpreted as patient effective dose or operator effective dose. Transmitted photon energy was scored in 1 keV bins. Spatial photon current and deposited energy were scored on a 50 × 50 grid over the 10 × 10 ${\mathrm{cm}}^{2}$ detector plane, giving 0.2 × 0.2 ${\mathrm{cm}}^{2}$ pixels. The central 2 × 2 ${\mathrm{cm}}^{2}$ region of interest (ROI) was used to quantify defect-associated hot spots.

For the main diagnostic-spectrum study, $1.0\times 10^{6}$ source histories were transported per case. Dedicated local-defect calculations used $5.0\times 10^{6}$ histories per condition (flat, 20% thinning, and 50% thinning) at all four tube potentials: 60, 80, 100, and 120 kVp. Transmission uncertainties were obtained from photon-counting statistics. For the high-statistics ROI dose, each primary history contributed one accumulated ROI-deposited-energy value (including zero for histories with no ROI deposition); the sample standard deviation over primary histories divided by the square root of the number of histories yielded the standard error of the per-source dose. Independent flat and defect standard errors were propagated to the dose-HEF ratio. Pixel-to-pixel spatial variation was not used as an uncertainty estimate; the reported dose-HEF uncertainty is based on event-by-event deposited-energy statistics. The dedicated high-statistics runs provide the ROI quantities reported in the Results section. Full 50 × 50 scoring maps were retained. The two-dimensional illustration is presented later in the Results section. That illustration uses the 5-million-history 60 kVp maps; adjoining 2 × 2 pixels were aggregated only for display. Quantitative inference relies on event-scored ROI averages rather than individual ratio-map pixels.

### 2.4. Attenuation Validation and Independent PHITS Verification

The material definition was first checked against Phy-X/PSD attenuation coefficients [23]; the NIST XCOM photon cross-section database provides the established tabulated reference framework for photon attenuation in elements, compounds, and mixtures [24]. For a 2.0 mm slab, the Geant4 uncollided transmission Tunc was converted to a mass attenuation coefficient by

(3) $${\left(\frac{\mu }{\rho }\right)}_{G4}=-\frac{\ln(T_{\mathrm{unc}})}{\rho t}.$$

Exact comparisons were made at 30, 40, 50, 60, 80, and 100 keV. The 30, 40, 50, and 100 keV points used $10^{6}$ histories; the 60 and 80 keV values were obtained from the matched benchmark runs. A 120 keV Phy-X value obtained by log–log interpolation between tabulated points is shown only as supplementary context and is not treated as an exact validation datum. To directly test the Bi K-edge, additional $10^{6}$-history, 2.0 mm flat-shield Geant4 runs were performed at 90, 91, 92, and 95 keV. These were compared against independently calculated NIST XCOM total mass attenuation coefficients including coherent scattering for the same elemental composition; Phy-X custom-energy K-edge values were not used. The 90/91 keV pair brackets the approximately 90.5-keV K-edge, while the two higher energies test the post-edge trend.

A second, independent code-to-code verification was performed using PHITS version 3.36 (Japan Atomic Energy Agency, Tokai, Ibaraki, Japan) [25]. Fifteen shielded configurations were matched between PHITS and Geant4 at monoenergetic photon energies of 60, 80, and 120 keV: flat, 20% strain, 50% strain, 20% local thinning, and 50% local thinning. Each benchmark used 100,000 source histories. Photon transmission was selected as the primary inter-code metric because the two implementations used different dose-transport/scoring details; absolute dose was therefore not used as the acceptance criterion for code agreement. The original attenuation check and the independent transmission benchmark are illustrated in Figure 2 and Figure 3, respectively.

### 2.5. Diagnostic X-Ray Spectra

Realistic tube spectra were generated with SpekPy version 2.5.4 (open-source software) [26]. A tungsten target, $12^{\circ }$ anode angle, 0.5 keV spectrum-bin width, and 2.5 mm added Al filtration were used. Tube potentials of 60, 80, 100, and 120 kVp were simulated. Each spectrum was normalized to unit integrated photon weight before sampling, so the reported quantities are per source photon rather than per mAs. Table 3 lists the spectrum descriptors returned by SpekPy; Figure 4 displays the normalized incident spectra.

### 2.6. Analytical Reference for Uniform Strain

Uniform thinning does not require Monte Carlo transport to predict the primary, uncollided component when material composition and density are unchanged. Therefore, a Beer–Lambert reference was calculated as

(4) $$T_{\mathrm{BL}}\left(\varepsilon ,E\right)=\exp\;\left[-\left(\frac{\mu }{\rho }\right)\left(E\right)\rho \frac{t_{0}}{\sqrt{1+\varepsilon }}\right].$$

Comparisons were performed at 60 and 80 keV using exact Phy-X mass attenuation coefficients and at 120 keV only as supplementary context, because the available Phy-X value at that energy was interpolated. Agreement of Equation (4) with uncollided Monte Carlo transport was used to separate the analytically predictable mass-thickness effect from phenomena that require full transport, including scattered transmission, secondary fluorescence, polychromatic spectral reshaping, and local leakage.

### 2.7. Derived Performance Metrics

Whole-field transmission was calculated as $T=N_{\mathrm{cross}}/N_{\mathrm{source}}$ and radiation protection efficiency as RPE = (1−T)×100%. For each kVp, the dose degradation ratio was DDR = $D_{\mathrm{deformed}}/D_{\mathrm{flat}}$. For local defects, the hot-spot enhancement factor (HEF) was the mean central 2 × 2 ${\mathrm{cm}}^{2}$ dose or photon current in the defect case divided by the corresponding central value for the flat shield. For a ratio R=A/B, independent one-sigma uncertainties were propagated as $\sigma_{R}/R={\left[{\left(\sigma_{A}/A\right)}^{2}+{\left(\sigma_{B}/B\right)}^{2}\right]}^{1/2}$. No hypothesis-testing p-values were applied because this study evaluates deterministic geometries by Monte Carlo sampling; numerical uncertainty and independent verification are reported instead.

## 3. Results

### 3.1. Material Validation

Across the six exact reference energies, the Geant4-derived mass attenuation coefficients agreed closely with Phy-X. The mean absolute relative difference was 0.54%, and the maximum absolute difference was 1.56%, at 80 keV. The differences at 30, 40, 50, 60, 80, and 100 keV were +0.15%, +0.10%, −0.18%, −0.74%, −1.56%, and −0.50%, respectively. The corresponding one-sigma Monte Carlo uncertainty of the Geant4 mass attenuation coefficient ranged from approximately 0.003 to 0.022 ${\mathrm{cm}}^{2}$$g^{-1}$ (Table 4). This verifies that the elemental composition, density, and uncollided-photon classification reproduce the expected tabulated attenuation behavior within the statistical precision and known cross-section-library differences of the calculation.

The additional XCOM comparison resolves the strong attenuation discontinuity close to the Bi K-edge (Table 5). The Geant4-derived coefficient increased from 0.7603±0.0015 ${\mathrm{cm}}^{2}$$g^{-1}$ at 90 keV to 2.6836±0.0035 ${\mathrm{cm}}^{2}$$g^{-1}$ at 91 keV. The corresponding XCOM values were 0.7828 and 2.7200 ${\mathrm{cm}}^{2}$$g^{-1}$; the Geant4-minus-XCOM differences across the four diagnostic energies were −2.87% to −0.78%. These systematic offsets are larger than the Monte Carlo counting uncertainties, so the comparison demonstrates reproduction of the K-edge structure, not agreement at the counting-statistical level.

### 3.2. PHITS-Geant4 Code Comparison

The two independent Monte Carlo codes reproduced the same deformation trends. Across the 15 matched shielded cases, the mean absolute relative transmission difference was 0.38% and the maximum was 0.81%. However, the mean combined one-sigma statistical precision of a matched PHITS-Geant4 pair was approximately 0.45% (about 0.40–0.53% across the benchmark). The observed 0.38% mean difference is therefore at the statistical floor of the benchmark and should be interpreted as consistency within Monte Carlo uncertainty, not as an intrinsic measurement of code agreement to 0.38%.

At 60 keV, for example, flat transmissions were 0.51856 (PHITS) and 0.52026 (Geant4); at 80 keV, they were 0.73260 and 0.73249; and at 120 keV, they were 0.70463 and 0.71034. The larger systematic offset at 120 keV remained below 1% and is small compared with the deformation effects studied subsequently. Because this benchmark was designed as an independent implementation and geometry check rather than as sub-percent cross-section metrology, its $10^{5}$ histories per case are interpreted only at their stated statistical precision; the production deformation effects are substantially larger than this numerical floor. Table 6 summarizes the matched comparisons.

### 3.3. Uniform Tensile Strain Under Realistic Spectra

Uniform strain progressively reduced the effective thickness and increased transmitted photon fluence at every tube potential (Figure 5). The effect was strongest for the softer spectrum. At 50% strain, transmission increased from 0.12186 to 0.16179 at 60 kVp, from 0.22914 to 0.27914 at 80 kVp, from 0.31467 to 0.36757 at 100 kVp, and from 0.36590 to 0.42018 at 120 kVp. These correspond to relative transmission increases of 32.8%, 21.8%, 16.8%, and 14.8%, respectively.

In protection-efficiency terms, 50% strain lowered RPE by 3.99, 5.00, 5.29, and 5.43 percentage points from 60 to 120 kVp. The apparent distinction between relative transmission increase and percentage-point RPE loss is important: when baseline transmission is low, even a moderate absolute increase in transmission can represent a large relative degradation of the transmitted field. The 50% strain dose ratios at 80 and 100 kVp—1.201±0.034 and 1.227±0.031—differ by only 0.026, which is smaller than their combined one-sigma uncertainty of approximately 0.046; the apparent ordering is therefore not interpreted as a statistically resolved reversal of the overall energy trend. The transmission and water-dose results are summarized in Table 7 and Figure 6, respectively.

### 3.4. Local Thinning: Whole-Field Metrics Versus Spatial Hot Spots

Because the central defect occupied only 4% of the shield area, whole-field transmission changed much less than in the uniform-strain geometry. At 50% local thickness loss, transmission increased by 15.0%, 9.23%, 6.36%, and 5.66% relative to the flat shield at 60, 80, 100, and 120 kVp, respectively (Figure 7). This averaging concealed a substantially larger regional loss of protection.

The dedicated 5-million-history ROI analysis confirmed that a local defect produces a much larger central response than whole-field averaging suggests (Figure 8). At 50% thickness loss, the event-scored dose HEFs were 2.435±0.106, 1.823±0.064, 1.508±0.048, and 1.395±0.042 at 60, 80, 100, and 120 kVp, respectively (Table 8). Dose errors are propagated from per-primary-history deposited-energy variation rather than from spatial pixel dispersion; the current HEFs have independent crossing-count uncertainties. The spatial maps and photon-current enhancement results are shown in Figure 9 and Figure 10, respectively.

Geometric sensitivity calculations at 60 kVp and 50% thinning (Table 9) confirmed an ROI–defect-area dependence. With the 2 cm ROI fixed, a 1 cm defect gave 1.341±0.107, while 2 and 4 cm defects gave 2.435±0.106 and 2.571±0.159. With the 2 cm defect fixed, the 1, 2, and 4 cm ROI results were 2.019±0.354, 2.435±0.106, and 1.404±0.075, respectively. The 1 cm ROI point has broad uncertainty and does not establish a spatial maximum. Varying the water-front position across 0.5, 1, and 5 cm gave 2.706±0.265, 2.435±0.106, and 2.609±0.288; no difference was statistically resolved within the present Monte Carlo precision. Neither this null resolution nor the central-defect geometry demonstrates distance independence or defect-position independence in a real garment.

### 3.5. Spectral Hardening and Energy Dependence

The flat ${\mathrm{Bi}}_{2}$$O_{3}$/TPU shield hardened all four source spectra. Using the SpekPy source means in Table 3, the mean photon energy increased from 35.95 to 45.46 keV at 60 kVp, from 42.90 to 54.89 keV at 80 kVp, from 49.14 to 61.81 keV at 100 kVp, and from 54.44 to 66.45 keV at 120 kVp. These shifts correspond to increases of approximately 26.5%, 28.0%, 25.8%, and 22.1%, respectively. Deformation then slightly modified the transmitted spectral distribution by changing the material path length. Figure 11 illustrates this behavior at 60 kVp for the flat, 50% strain, and 50% local-thinning cases.

For a quantitative comparison across all four tube potentials, Table 10 gives photon-count-weighted transmitted mean energies in each deformation state from the original $10^{6}$-history production spectra. The uniformly strained sheet softened the transmitted spectrum relative to the flat shield at every tube potential, whereas a 4%-area local defect produced smaller field-integrated changes. These values are descriptive spectral means, not substitutes for the full energy distributions.

The energy dependence should not be interpreted as a simple monotonic function of photon energy. Bismuth has a K absorption edge near 90.5 keV; consequently, monoenergetic attenuation and polychromatic response can change non-monotonically across this region. This is also why realistic spectrum transport is more informative than assigning one effective energy to each tube potential.

### 3.6. Beer–Lambert Reference Versus Monte Carlo Transport

For uniform strain, the uncollided component closely followed the analytical mass-thickness prediction. At the exact 60 and 80 keV reference energies, all 0, 20, and 50% strain cases showed small positive Geant4-minus-analytical residuals of approximately +0.39 to +0.69% (Table 11). This common sign is not treated as random scatter. At the same energies, the Geant4 mass attenuation coefficients were slightly below the Phy-X/XCOM-equivalent values, by 0.74% at 60 keV and 1.56% at 80 keV (Table 4); a slightly lower transport cross-section therefore predicts a slightly larger Geant4 uncollided transmission than a Beer–Lambert calculation based on the Phy-X coefficient. The residual pattern is consequently consistent with a small cross-section-library offset superposed on Monte Carlo counting uncertainty.

This comparison confirms that the primary effect of idealized uniform extension is the deterministic reduction in areal density. The value of Monte Carlo transport is instead in total transmitted fluence, including scatter, atomic relaxation, spectral reshaping, absorbed dose, and nonuniform geometries.

## 4. Discussion

### 4.1. Deformation-Induced Changes in Shielding Performance

The central numerical finding is that the protection predicted for a given shielding surrogate is geometry-dependent; no mechanical experiment was performed on a filled TPU sheet. A material can retain the same intrinsic elemental attenuation coefficient while its in-use shielding deteriorates because geometric deformation reduces the mass thickness presented to the beam. In the present idealized model, 50% uniform strain reduces the thickness from 2.0 to 1.633 mm. This produces a systematic rise in transmission across 60–120 kVp without requiring any change in material density or chemistry. Accordingly, flat-sheet μ/ρ, HVL, or attenuation efficiency alone cannot characterize the consequences of a prescribed change in mass thickness. Whether a particular filled elastomer undergoes that change must be established independently from composite-specific mechanical measurements.

The analytical comparison clarifies what is and is not novel in this observation. The primary uncollided component under uniform strain is almost completely described by Beer–Lambert attenuation once the deformed thickness is known. The contribution of Monte Carlo modeling is therefore not to rediscover exponential attenuation but to extend the analysis to realistic polychromatic spectra and quantities for which a one-dimensional slab equation is incomplete: total transmitted fluence, including scatter, spectral hardening and secondary emission, absorbed dose, and spatially localized leakage.

### 4.2. Local Defects, Garment Quality Assurance, and Whole-Area Dilution

The local-thinning calculations provide the clearest practical case for spatial scoring. A 2 × 2 ${\mathrm{cm}}^{2}$ defect occupies only 4% of the modeled sheet. Consequently, whole-field transmission at 50% thickness loss rose by 5.66–15.0% depending on kVp, while the central dose increased by factors of 1.39–2.44. The additional 1–4 cm defect/ROI sensitivity demonstrates that the numerical HEF also depends on how the averaging region overlaps the prescribed loss of thickness; the reference 4% defect area is one illustrative configuration, not a universal damage size.

The result connects directly to established quality-assurance practice for personal radiation-protective equipment. Lambert and McKeon introduced a dose- and location-based rationale for evaluating lead-apron defects, and later practical models and longitudinal programs have reinforced the importance of both defect position and area [12,13,14]. EURADOS guidance summarizes example maximum tolerable defect areas of 15 ${\mathrm{mm}}^{2}$ over critical organs and 670 ${\mathrm{mm}}^{2}$ clearly away from critical organs, while emphasizing regular inspection and local policy [27]. The 400 ${\mathrm{mm}}^{2}$ patch used here lies between those historical example areas; it is far larger than a critical-organ tolerance but below the quoted noncritical-area value. This comparison is contextual only: the present 2 mm ${\mathrm{Bi}}_{2}$$O_{3}$/TPU computational surrogate is not a tested protective garment; the idealized thickness step cannot represent apron cracks, seams, debonding, filler redistribution, or realistic wear, and its 400 ${\mathrm{mm}}^{2}$ area is not a clinical rejection threshold.

Recent evidence is increasingly dosimetric rather than purely geometric. A 2026 fluoroscopy study found that apron defects can meaningfully increase local air kerma and argued that defect location should be incorporated into rejection criteria [15]. Hanada et al. linked measured transmitted dose to defect area and, for their specific apron, exposure assumptions, and regulatory criterion, obtained a slit-defect discard threshold of 2.87 ${\mathrm{cm}}^{2}$ [16]. These values are necessarily protocol- and product-dependent, but they reinforce the central implication of the present simulation: local dose consequence, not whole-garment average transmission alone, is the physically relevant quantity when defects are spatially concentrated.

For future quality-assurance studies, defect area, position, averaging area, and detector separation should be reported explicitly. The present calculations demonstrate these dependencies in a simplified parallel-beam geometry and cannot be directly converted into patient or occupational dose or garment discard criteria.

### 4.3. Energy Dependence and Relation to Previous Flexible Shielding Studies

The stronger relative deformation effect at 60 kVp arises because the undeformed 2 mm composite attenuates the softer spectrum more strongly; a reduction in thickness therefore produces a larger fractional change in the transmitted field. At higher kVp, the flat shield is already more transmissive, so the same geometric thinning produces a smaller relative transmission increase. Bismuth K-edge structure further complicates simple energy ranking, particularly as the 100 and 120 kVp spectra extend above ~90.5 keV.

Previous work has demonstrated the feasibility of flexible Bi-containing shields in several polymer matrices. Cheraghi et al. reported lightweight flexible ${\mathrm{Bi}}_{2}$$O_{3}$ composites [5]; Koyuncu et al. developed ${\mathrm{Bi}}_{2}$$O_{3}$/waterborne-polyurethane coated fabrics [6]; Demirel and Yücel characterized flexible ${\mathrm{Bi}}_{2}$$O_{3}$/silicone composites [7]; and Dincer et al. reported Bi-Sn/TPU sheets [8]. Wu et al. also developed stretchable TPU composites with a Bi-containing low-melting alloy [9]. These studies establish the materials basis for flexible radiation protection. The present work addresses a different question: given a flexible shielding material, how does prescribed macroscopic deformation translate into spatially resolved in-use transmission and dose?

Recent reviews increasingly emphasize structure–mechanism–performance relationships, mechanical reliability, and predictive modeling for polymer shielding [1,2,3,4]. The framework presented here contributes to that direction by treating deformation geometry as an explicit radiation-transport input rather than as a qualitative descriptor of flexibility.

### 4.4. Verification Strength and Interpretation of the Monte Carlo Results

The numerical workflow uses two complementary verification levels. Agreement with Phy-X over the original six energies and with XCOM across the Bi K-edge demonstrates that the modeled elemental mixture reproduces the tabulated narrow-beam attenuation structure to the reported numerical differences. Independent PHITS calculations then show that the deformed geometries produce consistent total transmission in a second Monte Carlo code. Because the observed 0.38% mean absolute inter-code difference is comparable to the approximately 0.45% mean combined statistical precision, the benchmark supports consistency within Monte Carlo uncertainty rather than an intrinsic code-agreement accuracy of 0.38%. The deformation effects emphasized in the production study are much larger than this numerical floor, reducing the likelihood that the reported trends originate from a code-specific implementation error.

Absolute water dose was not used for PHITS-Geant4 validation because the two benchmark implementations did not use strictly identical dose-transport/scoring treatments. Consequently, there is no independent PHITS verification of the local ROI dose HEFs; these values are internally reproducible Geant4 event-scored results conditional on the Geant4 water-deposition implementation and simplified geometry. This is a radiation-transport verification, not a validation of a fabricated composite’s mechanical or shielding performance.

### 4.5. Limitations

The present model was designed to isolate the radiation-transport consequences of geometric deformation rather than to reproduce the full mechanical response of a specific ${\mathrm{Bi}}_{2}$$O_{3}$/TPU composite. Deformation was therefore prescribed kinematically, without solving a constitutive TPU model. Near-incompressibility is a reasonable first-order approximation for neat TPU [19], although the addition of 40 wt% ${\mathrm{Bi}}_{2}$$O_{3}$ may modify transverse contraction, stiffness, and damage evolution. The constant-volume deformation should consequently be interpreted as a controlled geometric assumption rather than a constitutive prediction. The illustrative ν=0.40–0.50 sensitivity demonstrates the influence of alternative prescribed transverse contractions while conserving shield mass; none of those values has been measured for this formulation.

The filler distribution was assumed to remain uniform during deformation, and strain-induced voiding, particle debonding, agglomeration, cracking, permanent set, and filler migration were not included. The elemental TPU composition was likewise used as a representative radiation-transport surrogate rather than as the formulation of a specific commercial grade. Local damage was represented by a reproducible sharp-edged region of reduced thickness. The sensitivity analysis varied central square-defect side length from 1 to 4 cm, but it did not test off-center locations, cracks, seams, delamination, or variable-depth wear morphology.

The irradiation geometry was intentionally simplified to a normally incident parallel X-ray field in vacuum with a thin downstream water scoring slab. Oblique incidence, surrounding-air and body scatter, curved anatomy, seams, and multilayer garment structures were outside the present scope. The reported dose values should therefore be interpreted as relative indicators of prescribed geometric changes rather than as direct estimates of patient or occupational dose. The detector-distance comparison used only three positions and did not resolve a change at the reported statistical precision; this does not establish spacing independence in a garment or clinical environment.

## 5. Conclusions

The Geant4 model of a 60 wt% TPU/40 wt% ${\mathrm{Bi}}_{2}$$O_{3}$ computational surrogate reproduces Phy-X mass attenuation coefficients at six energies (30–100 keV) and the Bi K-edge discontinuity given by XCOM, with systematic Geant4–XCOM offsets of approximately −0.8% to −2.9% near the edge. The PHITS–Geant4 verification establishes consistency of the matched photon-transmission observable within its reported statistical precision, but it does not independently validate local water-dose scoring or the mechanical performance of a real filled composite.

Under prescribed constant-volume uniform extension, 50% strain reduced thickness from 2.0 to 1.633 mm and increased whole-field transmission by 14.8–32.8% over 60–120 kVp. Beer–Lambert attenuation explained the primary areal-density effect. An illustrative mass-conserving transverse-response sensitivity changed the predicted 60 keV uncollided transmission at 50% extension from 0.5497 to 0.5629 across ν=0.50–0.40; these values are not measurements of composite Poisson behavior.

For a prescribed 50% local thickness loss in a central 2 × 2 ${\mathrm{cm}}^{2}$ patch, whole-field transmission increased by 5.66–15.0%, whereas event-scored central dose HEFs were 2.435±0.106, 1.823±0.064, 1.508±0.048, and 1.395±0.042 at 60, 80, 100, and 120 kVp. Additional calculations showed that the reported enhancement depends on defect size and ROI area. A three-position detector study did not statistically resolve HEF changes in this simplified setup, which does not establish general distance independence. These findings quantify limitations of spatial averaging within a specified transport geometry, not real-world garment durability, clinical dose, or acceptability thresholds.

## Figures and Tables

**Figure 1:**
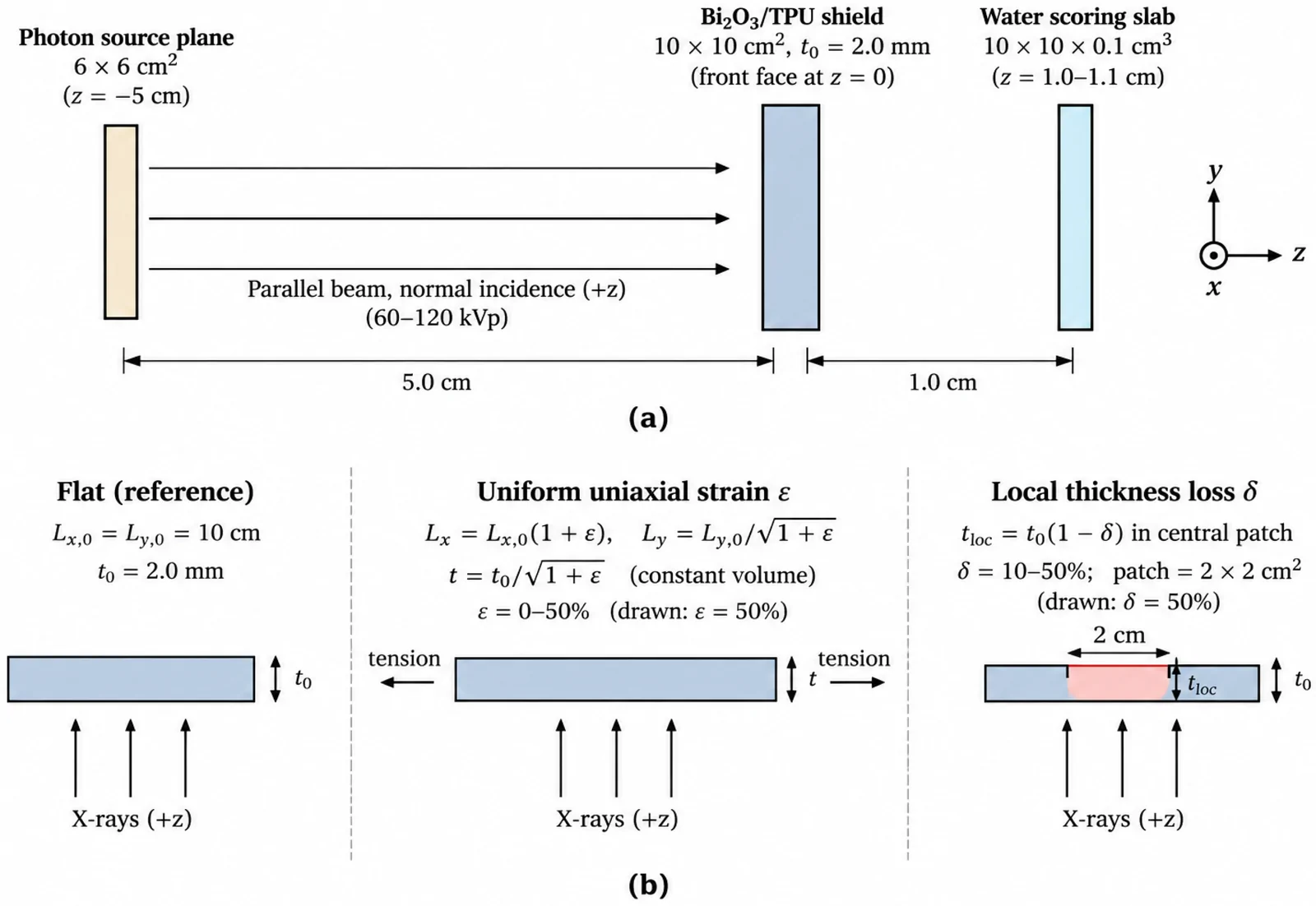
Monte Carlo simulation geometry and shield configurations. (**a**) Side-view radiation-transport geometry showing the 6 × 6 ${\mathrm{cm}}^{2}$ parallel X-ray source plane, the ${\mathrm{Bi}}_{2}$$O_{3}$/TPU composite shield with nominal dimensions of 10 × 10 ${\mathrm{cm}}^{2}$ and thickness $t_{0}=2.0$ mm, and the downstream 10 × 10 × 0.1 ${\mathrm{cm}}^{3}$ water scoring slab. (**b**) Cross-sectional representations of the flat reference shield, uniform uniaxial tensile strain ε, and localized thickness loss δ in a central 2 × 2 ${\mathrm{cm}}^{2}$ region. Under the constant-volume deformation assumption, the strained dimensions are defined by $L_{x}=L_{x,0}\left(1+\varepsilon \right)$, $L_{y}=L_{y,0}/\sqrt{1+\varepsilon }$, and $t=t_{0}/\sqrt{1+\varepsilon }$, whereas the local defect thickness is $t_{\mathrm{loc}}=t_{0}\left(1-\delta \right)$.

**Figure 2:**
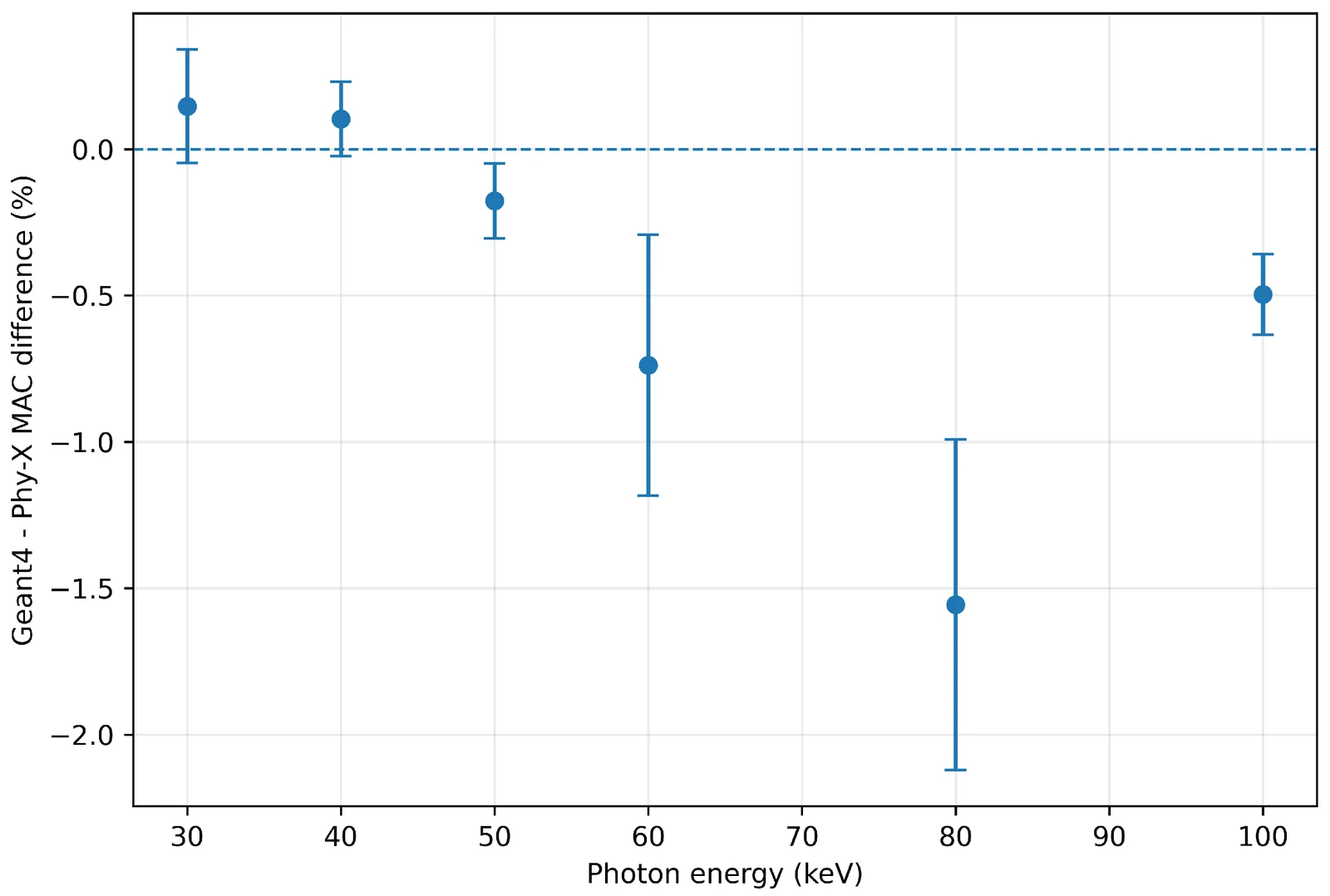
Percent difference between Geant4-derived and exact Phy-X mass attenuation coefficients. Error bars are one-sigma Monte Carlo uncertainties propagated from uncollided photon-counting statistics. The interpolated 120 keV reference is excluded. The dashed horizontal line indicates zero difference (exact numerical agreement).

**Figure 3:**
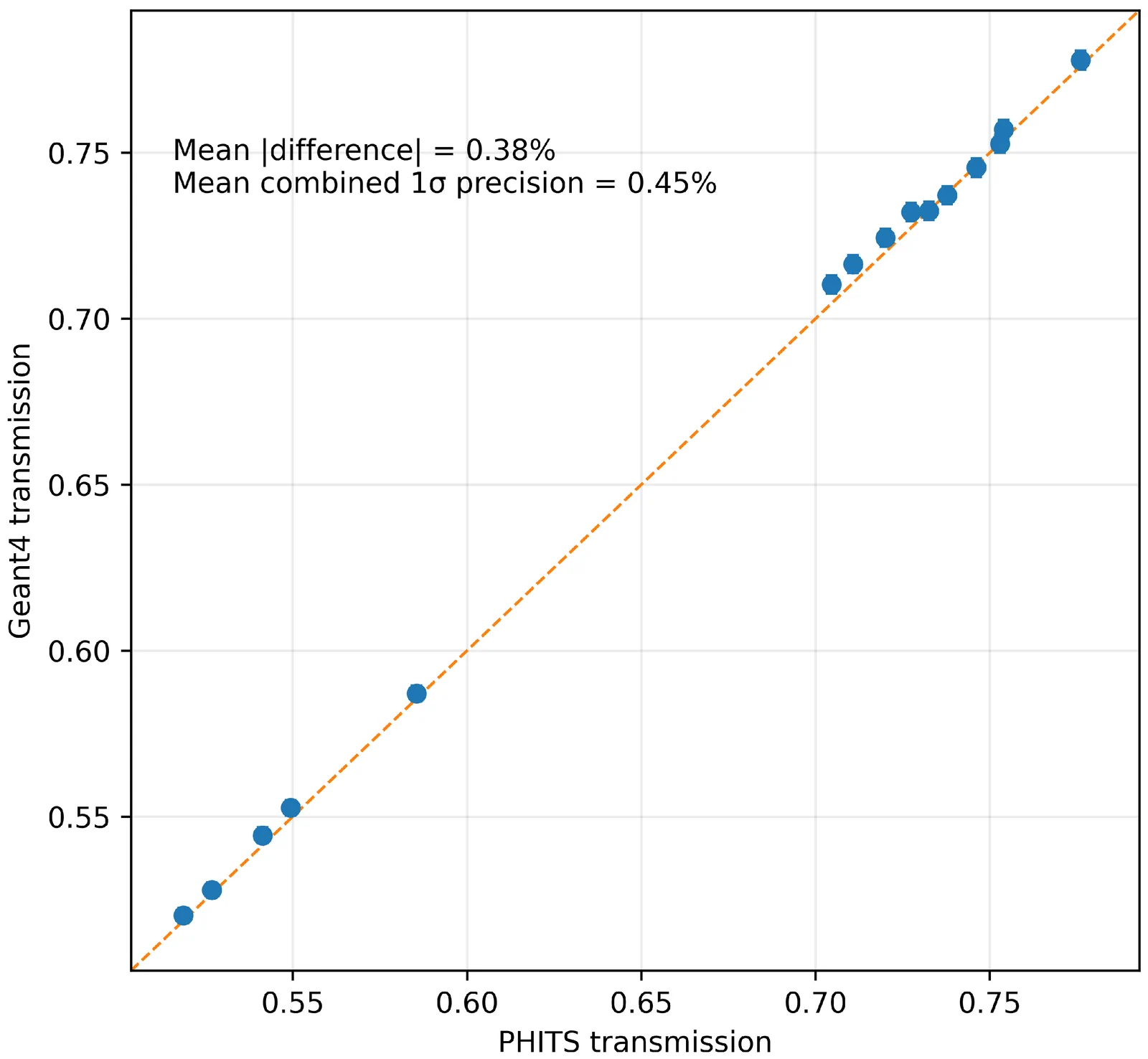
Independent PHITS–Geant4 comparison for 15 matched shielded cases at 60, 80, and 120 keV. Error bars show the reported one-sigma Monte Carlo precision of each code; the dashed line represents perfect agreement.

**Figure 4:**
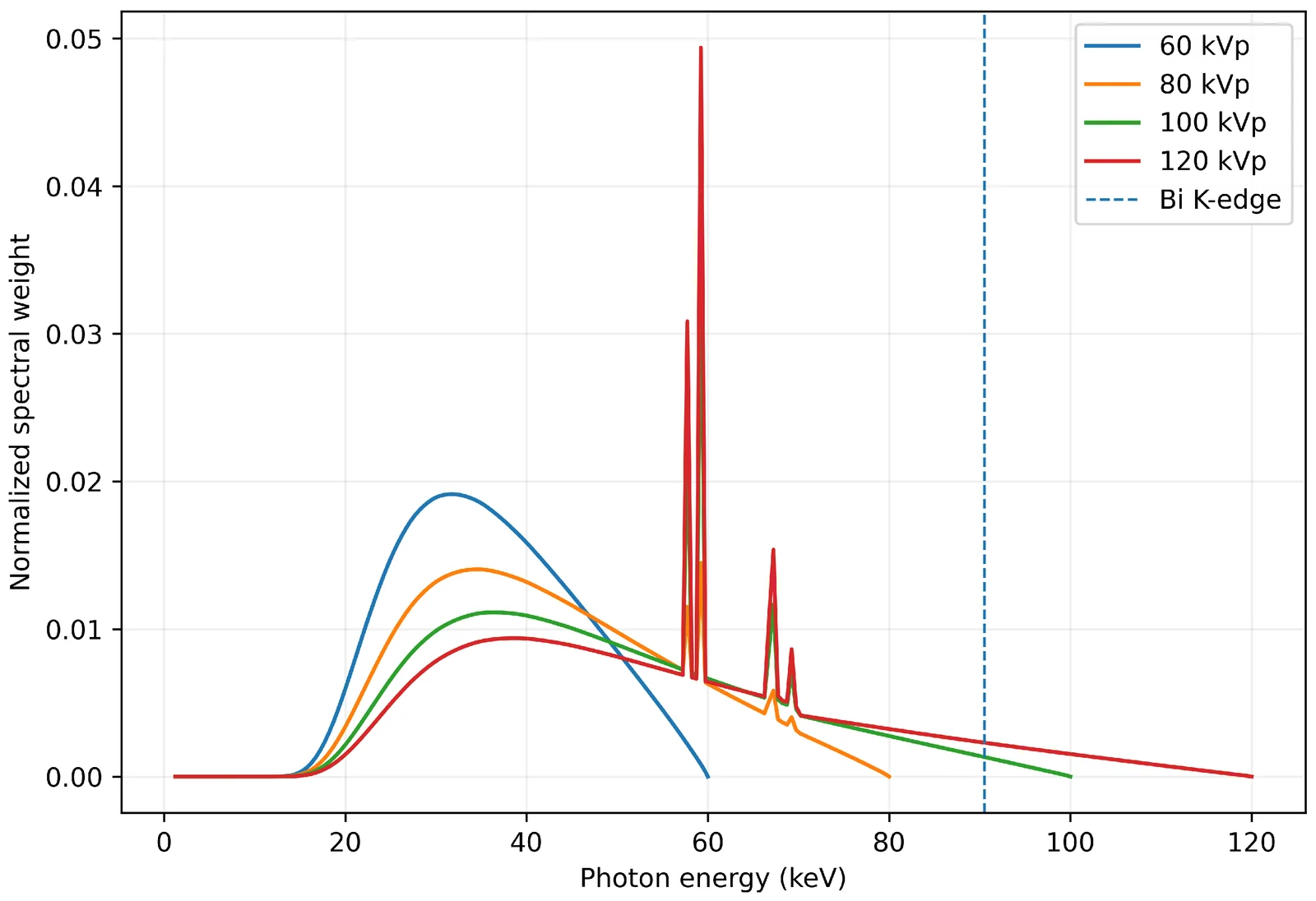
Normalized 60, 80, 100, and 120 kVp tungsten spectra used as source distributions. The vertical marker identifies the Bi K-edge (~90.5 keV), which contributes to the non-monotonic energy dependence of bismuth attenuation.

**Figure 5:**
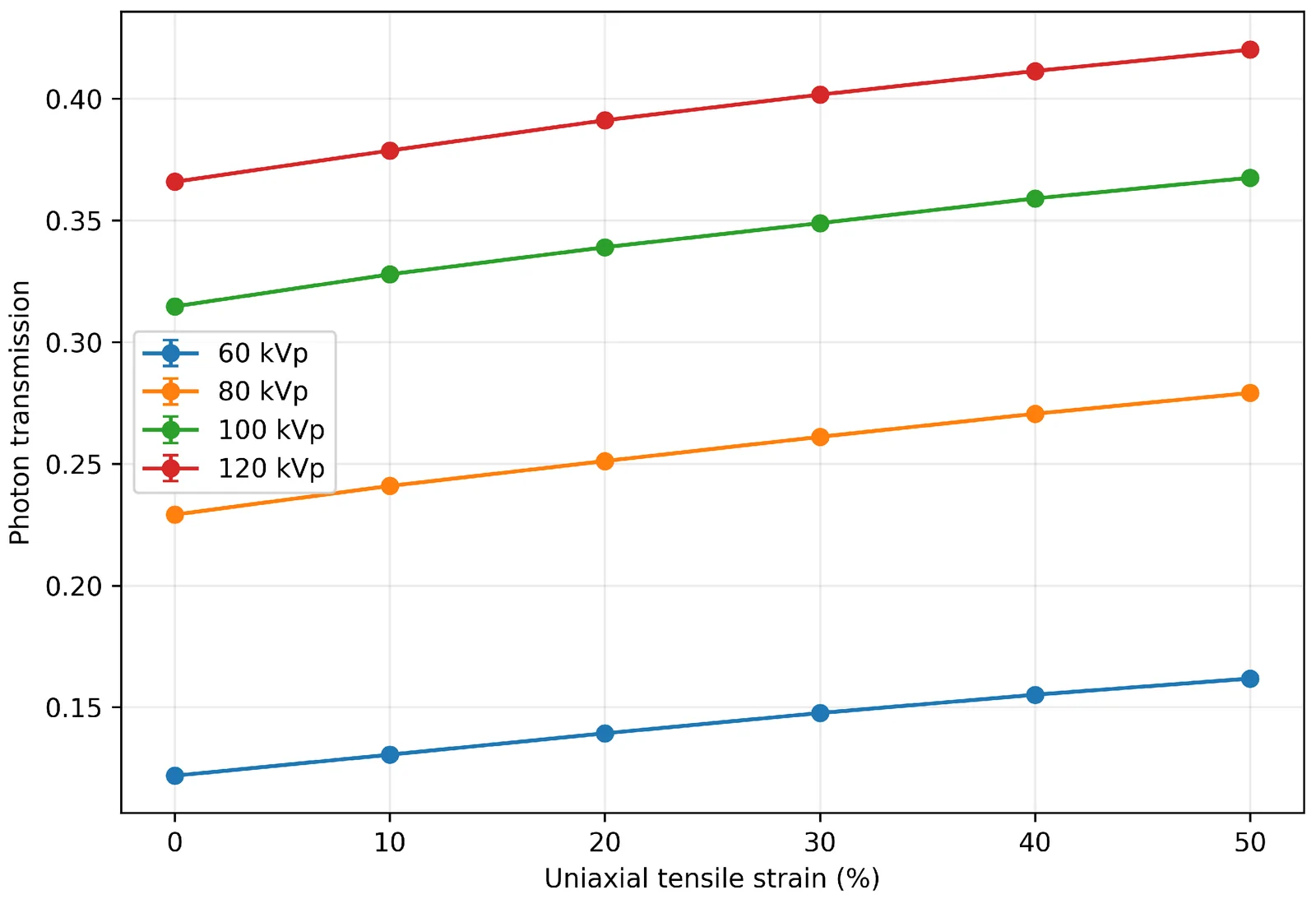
Photon transmission as a function of prescribed uniaxial strain for the four diagnostic spectra. Error bars are one-sigma photon-counting uncertainties and are smaller than many plot markers. The thickness reduction follows the constant-volume relation in Equation (1).

**Figure 6:**
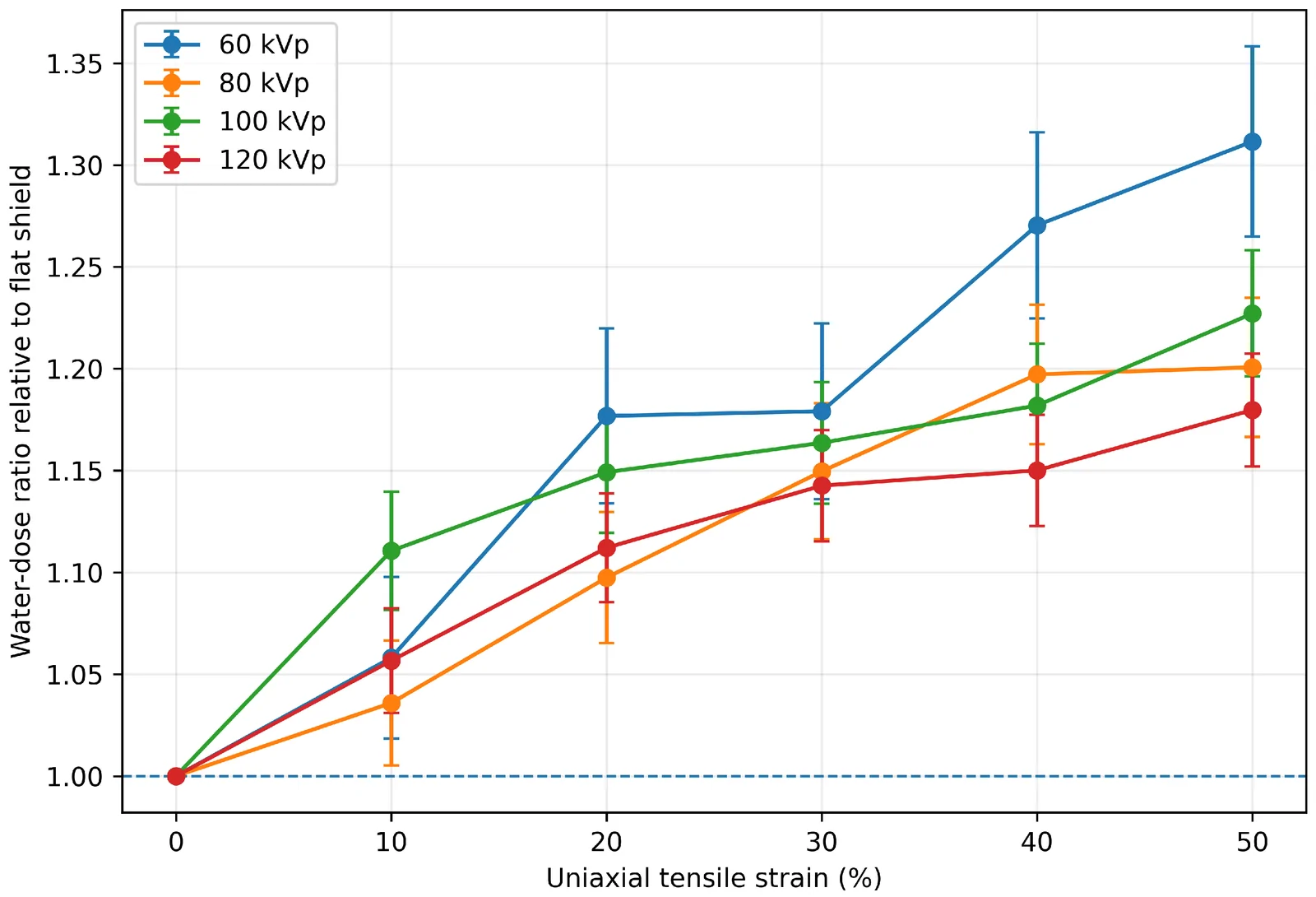
Whole-water-detector dose ratio relative to the flat shield as a function of strain. Error bars are one-sigma uncertainties propagated from the event-by-event dose standard errors of the flat and deformed cases. The non-monotonic point-to-point fluctuations are consistent with the larger dose-scoring uncertainty and are not interpreted as physical reversals. The dashed horizontal line marks a dose ratio of unity (flat-shield reference).

**Figure 7:**
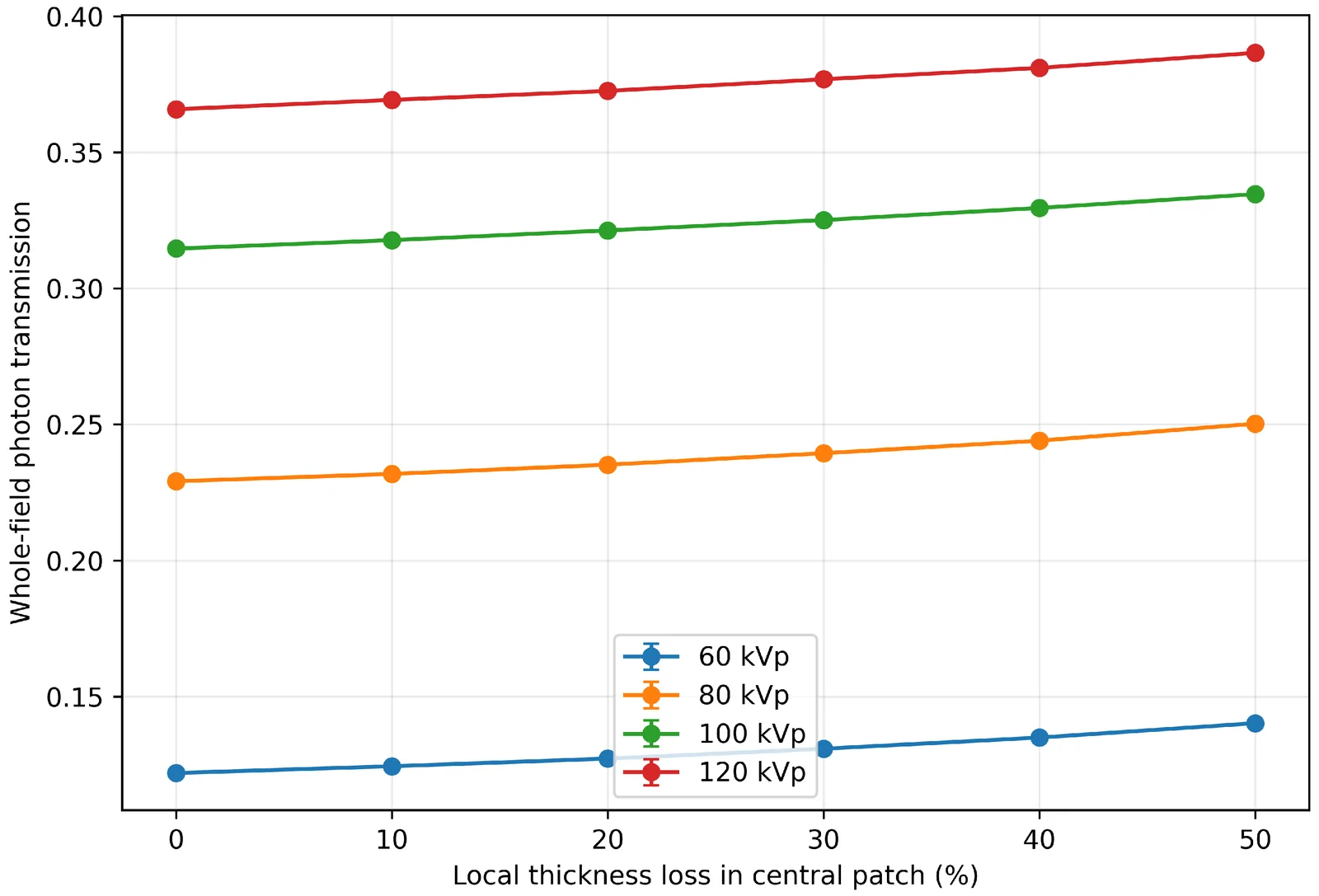
Whole-field transmission versus local thickness loss in the central 2 × 2 ${\mathrm{cm}}^{2}$ patch. Error bars are one-sigma photon-counting uncertainties. The limited area of the defect strongly dilutes its effect on the whole-detector average.

**Figure 8:**
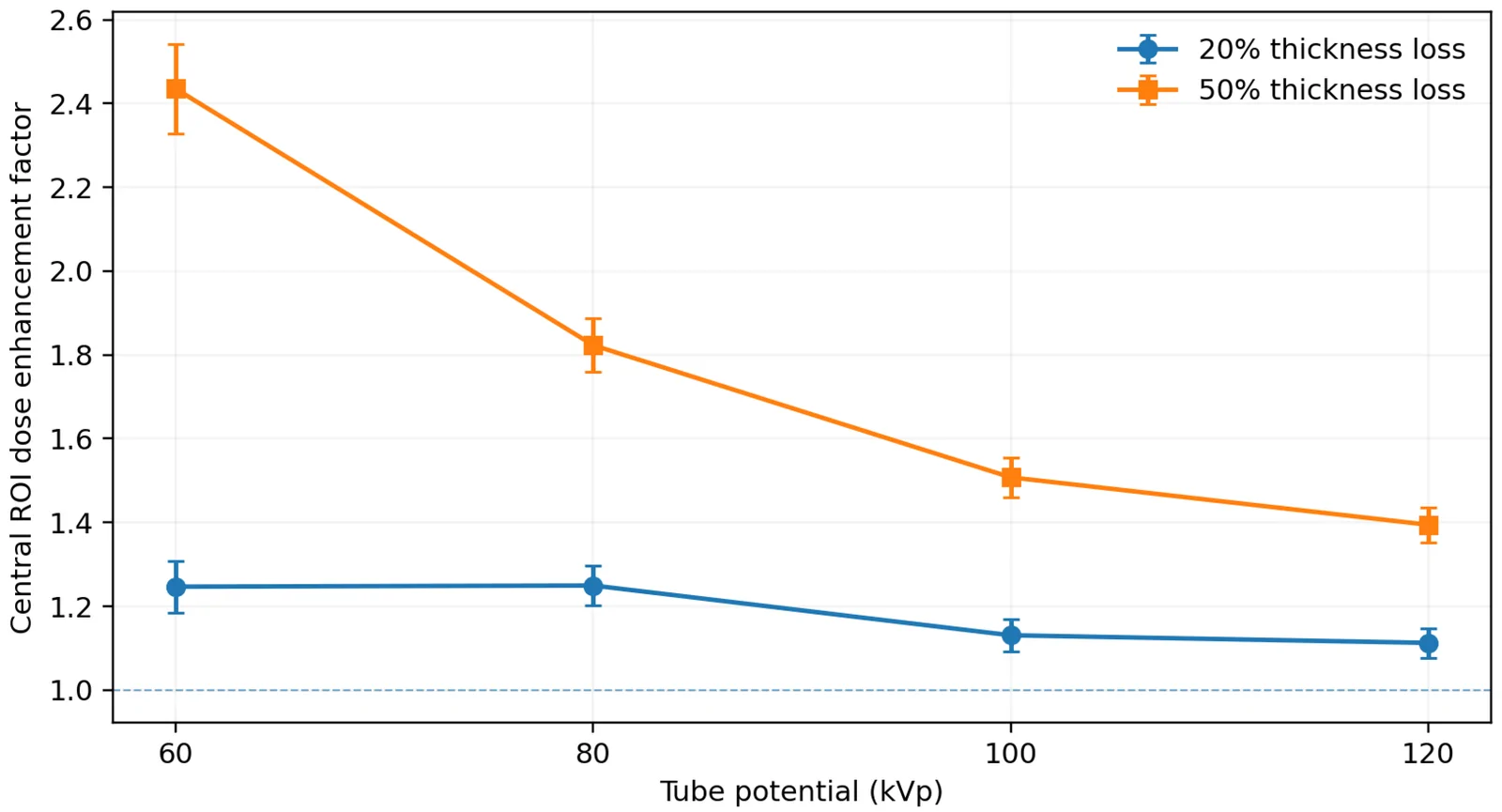
High-statistics central dose hot-spot enhancement factors for 20% and 50% local thickness loss at 60, 80, 100, and 120 kVp. Error bars represent event-based one-sigma uncertainties. The dashed horizontal line marks HEF = 1 (no enhancement relative to the flat shield).

**Figure 9:**
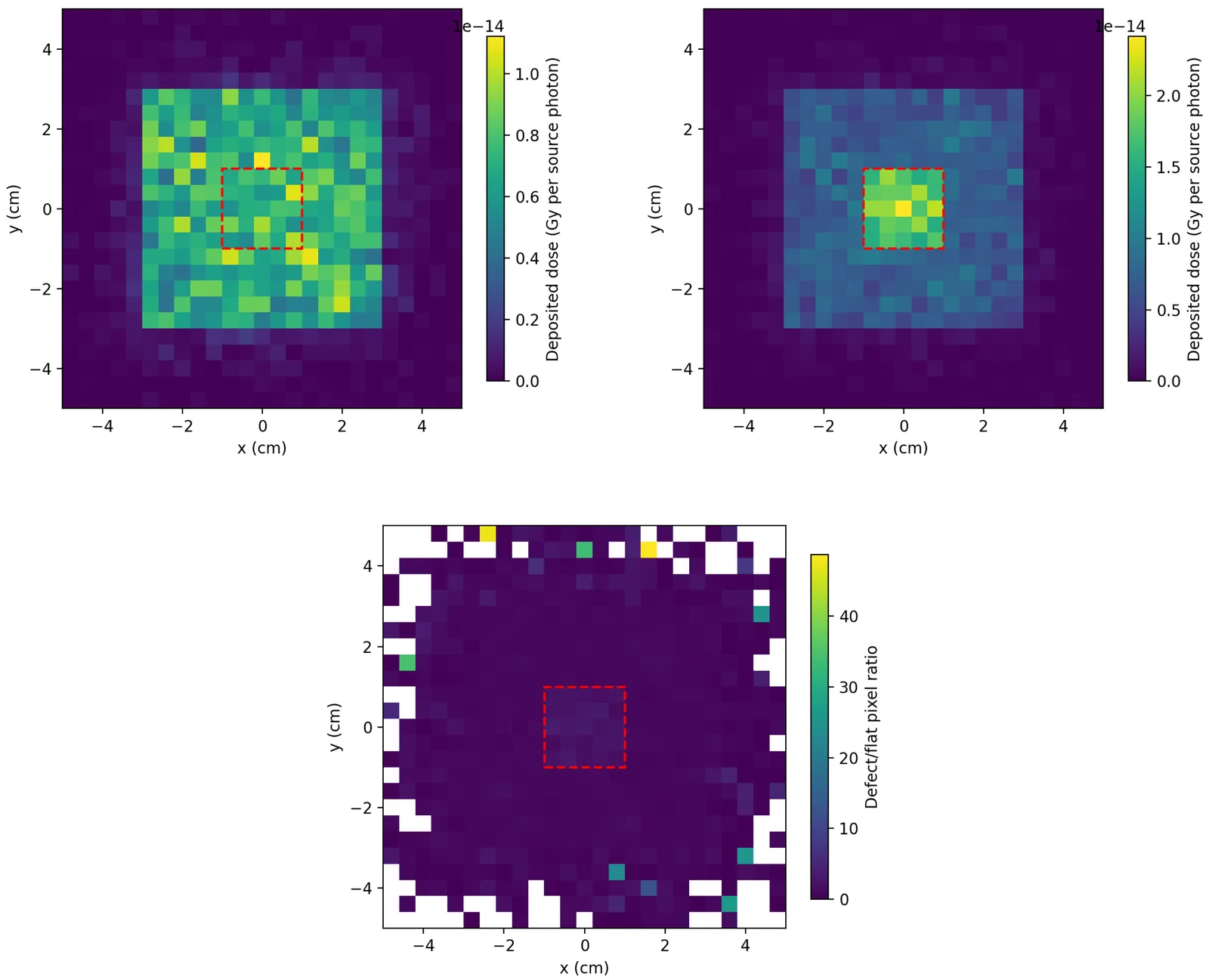
Two-dimensional water-dose scoring at 60 kVp from the 5-million-history high-statistics calculations: flat shield (**upper left**), 50% local thickness loss in the central 2 × 2 ${\mathrm{cm}}^{2}$ patch (**upper right**), and the corresponding local dose ratio (**lower panel**). The native 0.2 cm pixels were aggregated in 2 × 2 groups solely for display. The ratio map is qualitative: isolated high/low pixels can reflect sparse deposition, and quantitative comparisons use event-scored central-ROI dose HEFs (Table 8) rather than individual pixels. The red dashed square in each panel marks the central 2×2 ${\mathrm{cm}}^{2}$ defect/ROI; dose-map color bars use scientific notation.

**Figure 10:**
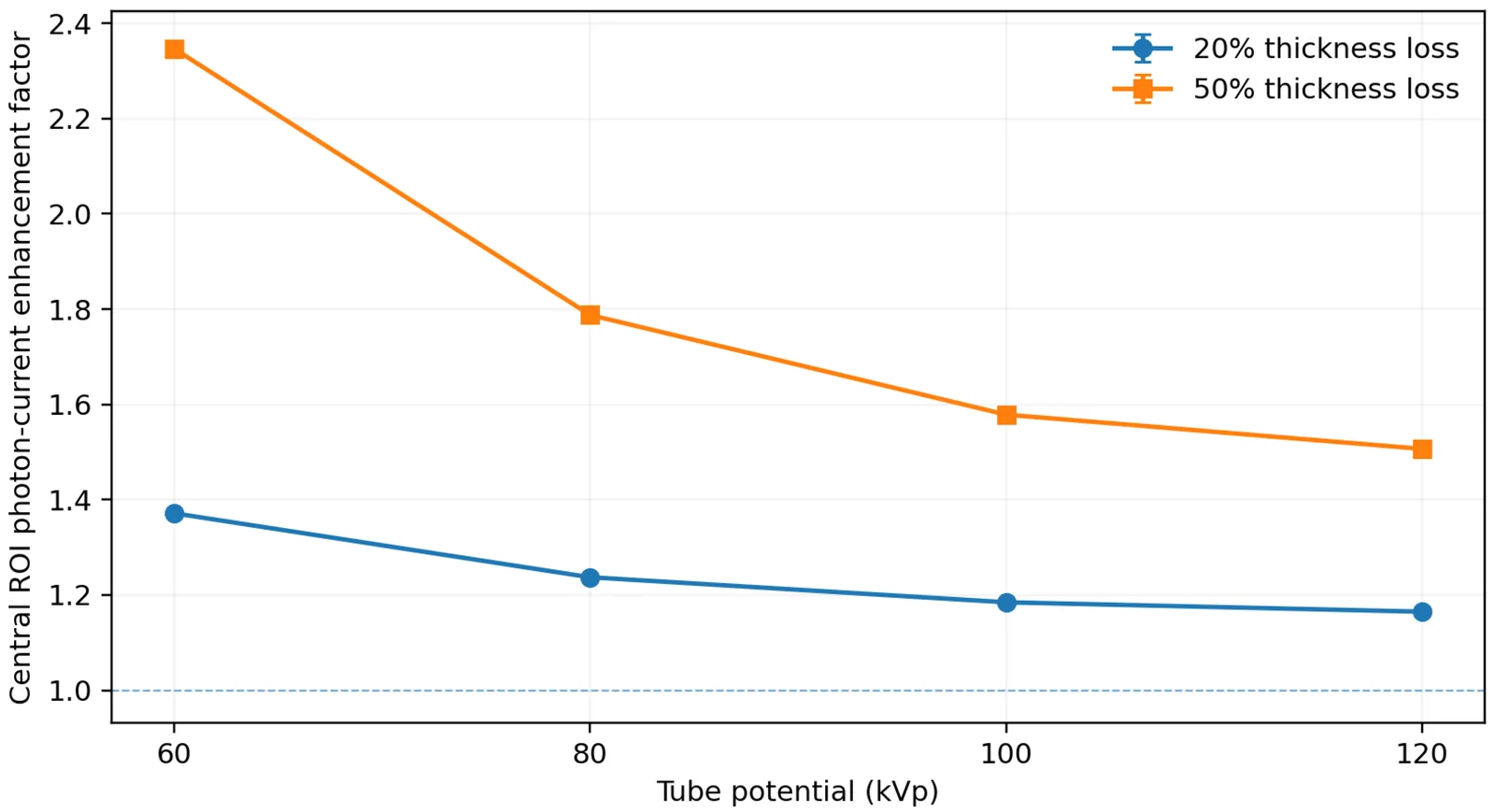
High-statistics central photon-current enhancement factors at 60, 80, 100, and 120 kVp from the same 5-million-history calculations as Table 8; one-sigma uncertainties are propagated from central crossing counts. The dashed horizontal line marks HEF = 1 (no enhancement relative to the flat shield).

**Figure 11:**
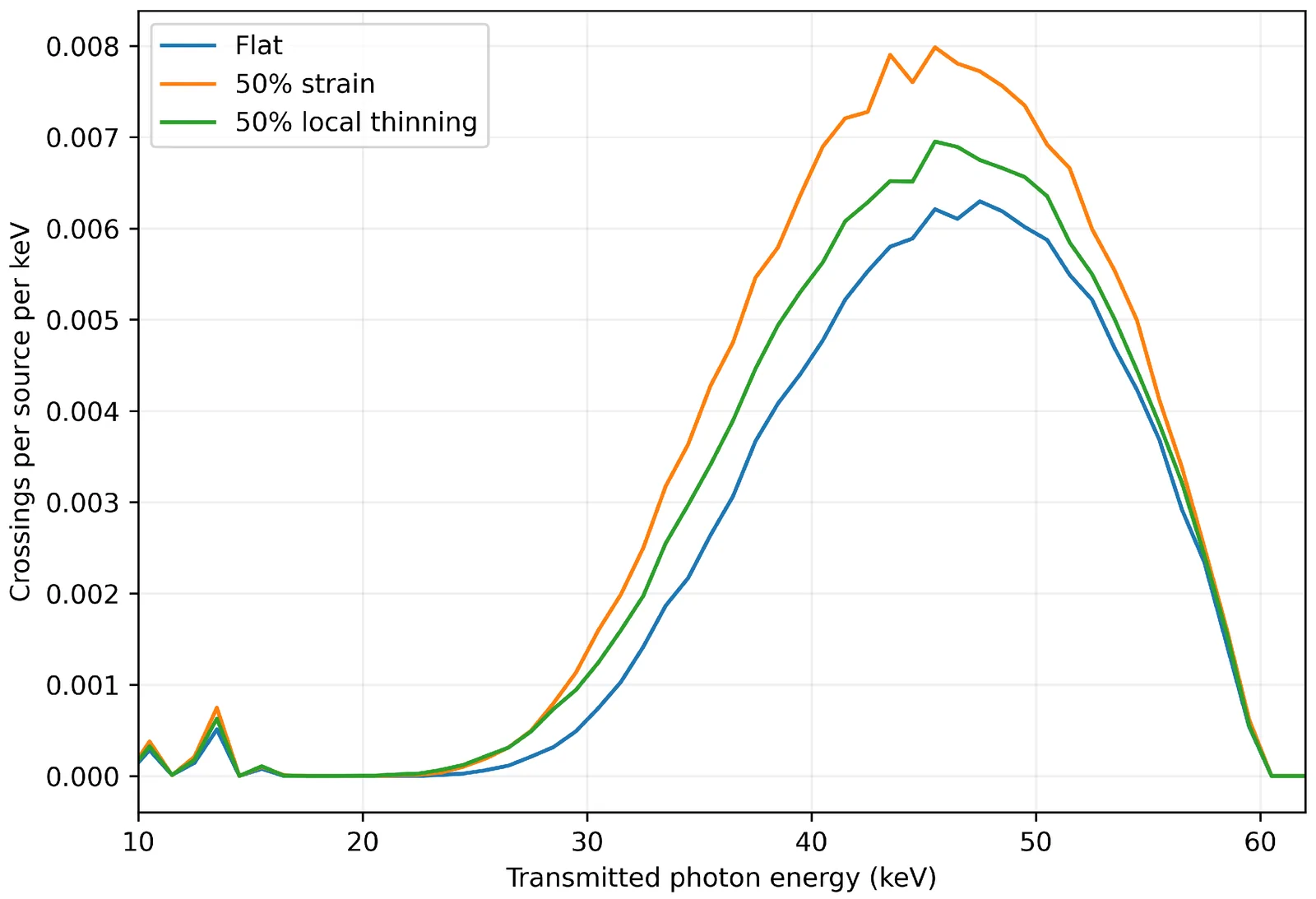
Transmitted 60 kVp spectra for the flat shield, 50% uniform strain, and 50% local thickness loss. Uniform deformation changes the path length across the entire field; local thinning increases leakage only within the defect region and, therefore, has a smaller effect on the field-integrated spectrum.

**Table 1:** Geometric dimensions of the prescribed constant-volume uniaxial strain model.

Strain (%)	Lx (cm)	Ly (cm)	Thickness (mm)
0	10.000	10.000	2.0000
10	11.000	9.535	1.9069
20	12.000	9.129	1.8257
30	13.000	8.771	1.7541
40	14.000	8.452	1.6903
50	15.000	8.165	1.6330

**Table 2:** Illustrative mass-conserving transverse-response sensitivity at 50% extension. The attenuation reference uses the existing exact 60 keV Phy-X mass attenuation coefficient; ν is not a measured composite property.

ν	Thickness (mm)	Density (g ${\mathrm{cm}}^{-3}$)	Beer–Lambert $T_{\mathrm{unc}}$ (60 keV)
0.40	1.7006	1.6921	0.562939
0.45	1.6664	1.7621	0.556353
0.48	1.6463	1.8055	0.552375
0.50	1.6330	1.8351	0.549712

**Table 3:** SpekPy spectrum characteristics used in the production simulations.

Tube Potential (kVp)	Mean Energy (keV)	First HVL in Al (mm)
60	35.95	2.082
80	42.90	2.734
100	49.14	3.458
120	54.44	4.219

**Table 4:** Exact Phy-X versus Geant4 mass attenuation coefficient validation. Geant4 uncertainties are one-sigma values propagated from uncollided counting statistics.

Energy (keV)	Phy-X μ/ρ (${\mathrm{cm}}^{2}$/g)	Geant4 μ/ρ (${\mathrm{cm}}^{2}$/g)	Difference (%)
30	11.48462	11.5014±0.0223	+0.15±0.19
40	5.50635	5.5120±0.0070	+0.10±0.13
50	3.13330	3.1278±0.0040	−0.18±0.13
60	1.99678	1.9820±0.0089	−0.74±0.45
80	1.01442	0.9986±0.0057	−1.56±0.57
100	2.15934	2.1486±0.0030	−0.50±0.14

**Table 5:** Geant4–XCOM mass attenuation comparison across the Bi K-edge. XCOM values refer to total attenuation including coherent scattering for the modeled elemental mixture; Geant4 uncertainties are one-sigma counting uncertainties.

Energy (keV)	XCOM (${\mathrm{cm}}^{2}$ $g^{-1}$)	Geant4 (${\mathrm{cm}}^{2}$ $g^{-1}$)	Difference (%)
90	0.7828	0.7603±0.0015	−2.87
91	2.7200	2.6836±0.0035	−1.34
92	2.6480	2.6185±0.0035	−1.11
95	2.4490	2.4300±0.0033	−0.78

**Table 6:** PHITS–Geant4 transmission benchmark over five deformation cases at each energy. The combined precision is the mean quadrature-combined one-sigma relative Monte Carlo uncertainty for the matched pairs.

Energy (keV)	Mean |ΔT| (%)	Maximum |ΔT| (%)	Mean Combined 1σ Precision (%)
60	0.390	0.588	0.517
80	0.089	0.240	0.408
120	0.653	0.810	0.419

**Table 7:** Effect of 50% uniform strain on whole-field transmission, protection efficiency, and water-dose ratio. Uncertainties are one-sigma Monte Carlo values; RPE uncertainty follows directly from transmission uncertainty, while DDR uncertainty is propagated from the flat and strained dose estimates.

kVp	*T* Flat	*T* at 50% Strain	RPE Flat/50% (%)	DDR at 50%
60	0.12186±0.00035	0.16179±0.00040	87.81/83.82	1.312±0.047
80	0.22914±0.00048	0.27914±0.00053	77.09/72.09	1.201±0.034
100	0.31467±0.00056	0.36757±0.00061	68.53/63.24	1.227±0.031
120	0.36590±0.00060	0.42018±0.00065	63.41/57.98	1.180±0.028

**Table 8:** Central 2 × 2 cm^2^ hot-spot enhancement factors from $5\times 10^{6}$ histories per case at all four kVp values. Dose uncertainties are event-based one-sigma standard errors propagated across independent flat and defect cases; current uncertainties are based on photon crossings.

kVp	Local Thickness Loss (%)	Dose HEF	Photon-Current HEF
60	20	1.247±0.061	1.371±0.007
60	50	2.435±0.106	2.347±0.011
80	20	1.250±0.048	1.237±0.005
80	50	1.823±0.064	1.788±0.006
100	20	1.131±0.038	1.185±0.004
100	50	1.508±0.048	1.579±0.005
120	20	1.113±0.035	1.165±0.004
120	50	1.395±0.042	1.507±0.004

**Table 9:** Geometric sensitivity of the dose HEF at 60 kVp and 50% thickness loss. The 2 cm defect/ROI and 1 cm water-front reference is from the independent 5-million-history case; other sensitivity cases use 1 million histories. Distances refer to shield-front-to-water-front *z* coordinates, not to the back-face air gap.

Varied Quantity	Value	Dose HEF ± SEM
ROI side (cm), defect 2 cm	1	2.019±0.354
	2	2.435±0.106
	4	1.404±0.075
Defect side (cm), ROI 2 cm	1	1.341±0.107
	2	2.435±0.106
	4	2.571±0.159
Water-front *z* (cm)	0.5	2.706±0.265
	1.0	2.435±0.106
	5.0	2.609±0.288

**Table 10:** Source and field-integrated transmitted mean photon energies (keV) calculated from the production energy-bin counts for each tube potential and geometric state.

kVp	Source	Flat Shield	50% Uniform Strain	50% Local Thinning
60	35.95	45.46	44.50	44.77
80	42.90	54.89	53.66	54.14
100	49.14	61.81	60.49	61.11
120	54.44	66.45	65.27	65.79

**Table 11:** Analytical versus Geant4 uncollided transmission at exact Phy-X reference energies. Geant4 uncertainties are one-sigma binomial counting uncertainties; the difference uncertainty is propagated relative to the fixed Beer–Lambert reference.

Energy (keV)	Strain (%)	t (mm)	Beer–Lambert T	G4 Uncollided T	Difference (%)
60	0	2.0000	0.480543	0.483150±0.001580	+0.543±0.329
60	20	1.8257	0.512227	0.515720±0.001580	+0.682±0.309
60	50	1.6330	0.549712	0.551860±0.001573	+0.391±0.286
80	0	2.0000	0.689145	0.693150±0.001458	+0.581±0.212
80	20	1.8257	0.711866	0.715350±0.001427	+0.489±0.200
80	50	1.6330	0.737872	0.742990±0.001382	+0.694±0.187

## Data Availability

The original contributions presented in this study are included in the article and Supplementary Materials. Supplementary File S1 contains the processed numerical datasets for attenuation validation, transmission benchmarking, uniform-strain and local-thinning analyses, event-based ROI results, geometric sensitivity analyses, Bi K-edge comparisons, source-spectrum metadata, and spatial map arrays. Further inquiries can be directed to the corresponding author.

## Supplementary Materials

The following supporting information can be downloaded at https://www.mdpi.com/article/10.3390/polym18192363/s1: Supplementary File S1 contains processed numerical datasets for attenuation validation, PHITS–Geant4 transmission benchmarking, Beer–Lambert comparisons, diagnostic-spectrum simulations, the four-kVp event-based ROI analysis, geometric sensitivity calculations, and Geant4–XCOM Bi K-edge comparisons, together with source-spectrum metadata and the 60 kVp spatial map arrays.
